# Automated Collateral Classification on CT Angiography in Acute Ischemic Stroke: Performance Trends Across Hyperparameter Combinations

**DOI:** 10.3390/bioengineering13010124

**Published:** 2026-01-21

**Authors:** Chi-Ming Ku, Tzong-Rong Ger

**Affiliations:** 1 Department of Biomedical Engineering, Chung Yuan Christian University, Taoyuan 320314, Taiwan; 2 Department of Biomedical Engineering, National Yang Ming Chiao Tung University, Taipei 112304, Taiwan

**Keywords:** collateral of acute ischemic stroke, CT angiography, vessel segmentation, collateral classification, hyperparameter analysis

## Abstract

Collateral status is an important therapeutic indicator for acute ischemic stroke (AIS), yet visual collateral grading remains subjective and suffers from inter-observer variability. To address this limitation, this study automatically extracted binarized vascular morphological features from CTA images and developed a convolutional neural network (CNN) for automated collateral classification. Performance trends were systematically analyzed across diverse hyperparameter combinations to meet different clinical decision needs. A total of 157 AIS patients (median age 65 [57–74] years; 61.8% were male) were retrospectively enrolled and stratified by Menon score into good (3–5, *n* = 117) and poor (0–2, *n* = 40) collateral groups. A total of 192 architectures were established, and three representative model tendencies emerged: a sensitivity-oriented model (AUC = 0.773; sensitivity = 87.18%; specificity = 65.00%), a balanced model (AUC = 0.768; sensitivity = 72.65%; specificity = 77.50%), and a specificity-oriented model (AUC = 0.753; sensitivity = 63.25%; specificity = 85.00%). These results demonstrate that kernel size, the number of filters in the first layer, and the number of convolutional layers are key determinants of performance directionality, allowing tailored model selection depending on clinical requirements. This work highlights the feasibility of CTA-based automated collateral classification and provides a systematic framework for developing models optimized for sensitivity, specificity, or balanced decision-making. The findings may serve as a reference for clinical model deployment and have potential for integration into multi-objective AI systems for endovascular thrombectomy patient triage.

## 1. Introduction

Endovascular thrombectomy (EVT) is the established treatment for acute ischemic stroke (AIS) [1], supported by multiple randomized trials demonstrating its benefit in appropriately selected patients [2,3,4,5,6]. However, clinical eligibility must be determined within a narrow therapeutic window, and accurate evaluation of salvageable tissue remains challenging. Among prognostic indicators, baseline collateral status plays a crucial role in predicting treatment outcome, infarct progression, and reperfusion benefit [7,8,9,10]. Computed tomography angiography (CTA) is widely available, rapid to acquire, and commonly used for collateral assessment in emergency settings [11], making it a practical tool for prompt clinical decision-making. Despite its clinical importance, the current collateral grading systems rely on limited visual classification criteria. This process is highly dependent on the radiologist’s clinical experience, resulting in poor inter-observer agreement and consuming valuable time during the management of AIS [12]. Thus, an objective, efficient, and automated CTA-based collateral assessment approach is urgently needed in clinical practice.

Conventional collateral scores are assessed using CTA, which rely on the morphologic extent of the collateral [13,14,15]. Most studies on collateral grading [16,17,18,19], as well as commercial software such as StrokeViewer [20,21], are based on the results of vessel segmentation. Convolutional neural networks (CNNs) have been widely used in the classification of radiological images [22,23,24,25]. Currently, there are automated collateral grading methods based on 3D CTA images and deep neural networks [26,27]; however, few studies have utilized CTA images for neural network-based automatic grading [28], and commercial software typically requires additional licensing fees. These factors will limit its applicability in medical institutions with limited resources. In addition, depending on the different clinical goals or the conservativeness of treatment decisions, there are different clinical requirements for sensitivity and specificity [29]. Therefore, the aim of this study is to automatically extract binarized vascular morphology from CTA and construct a CNN-based collateral classification framework while systematically investigating performance tendencies across multiple hyperparameter combinations to support different clinical decision-making scenarios.

## 2. Materials and Methods

### 2.1. Data Acquisition

This retrospective study was approved by the Ethics Committee of Chang Gung Memorial Hospital, Linkou Medical Center, Taiwan (IRB No.: 202500947B0), with informed consent waived due to anonymized data use. Imaging and clinical records of consecutive AIS patients who underwent mechanical thrombectomy for large-vessel occlusion of the anterior circulation between April 2015 and July 2019 were reviewed (initial cohort: *N* = 207). Demographic and clinical data were extracted from electronic medical records, including age, sex, baseline National Institutes of Health Stroke Scale (NIHSS) score, and 90-day modified Rankin Scale (mRS) score, while imaging data were obtained from Picture Archiving and Communication System (PACS). The inclusion criteria were adult patients who underwent baseline CTA scans. The exclusion criteria were patients with motion artifacts affecting image quality, patients with incomplete anatomical coverage, or patients with missing CTA series. After quality screening, 157 cases met the inclusion criteria and were analyzed in this study.

Collateral status was visually graded by two neuroradiologists (Dr. Chang, 20 years of neurology experience; Dr. Wu, 15 years of neuroradiology experience) using the 6-point Menon CTA scoring system [30]. Both raters were blinded to all clinical information and outcomes. Disagreements were resolved by consensus review. Patients were then categorized into two groups for model training and evaluation based on Bijoy K. Menon et al.’s criteria for discriminating clinical outcomes [30], reflecting clinically meaningful differences in outcome—good collateral status: Menon score 3–5 (*n* = 117) and poor collateral status: Menon score 0–2 (*n* = 40). This dataset served as the reference ground truth for subsequent image processing, model training, and hyperparameter analysis.

### 2.2. Image Preprocessing

In this study, the model was trained using binary regions of vascular morphological that were automatically extracted from the CTA maximum intensity projection image. Each patient was classified using the ganglionic level and the supraganglionic level. The image preprocessing pipeline was mainly based on our previous work [31], with minor modifications for the current dataset. To ensure reproducibility, the main procedures are briefly described below.

All processing was conducted on 8-bit grayscale BMP images converted from 0 to 450 Hounsfield units in DICOM images to assign the highest grayscale value to most of the cranium region [32]. The main processing flow was divided into brain region segmentation and vessel segmentation. For brain region segmentation, the original image was binarized based on the highest grayscale value of 255 to define the cranial region. We used the 8-connected flood fill algorithm to obtain the whole cranium with the brain region. Next, we performed the XOR operation on the cranium region image and the whole cranium with the brain region image to obtain the brain region images. Additionally, we removed the pixels close to the highest grayscale at the boundary of the brain and the cranium but not defined as cranium information using Sobel operator boundary detection, with G ≥ 255 applied three times on the brain region image. If the brain region included the background region due to the calvarial defect, the OPEN operation was performed until the brain region and background region were separated.

**Figure 1:**
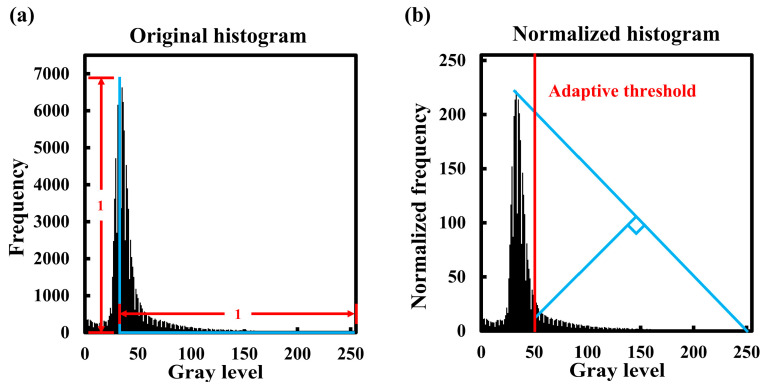
Schematic diagram of adaptive threshold calculation based on triangle method. (**a**) Histogram of original BMP image; (**b**) normalized histogram of BMP images.

**Figure 2:**
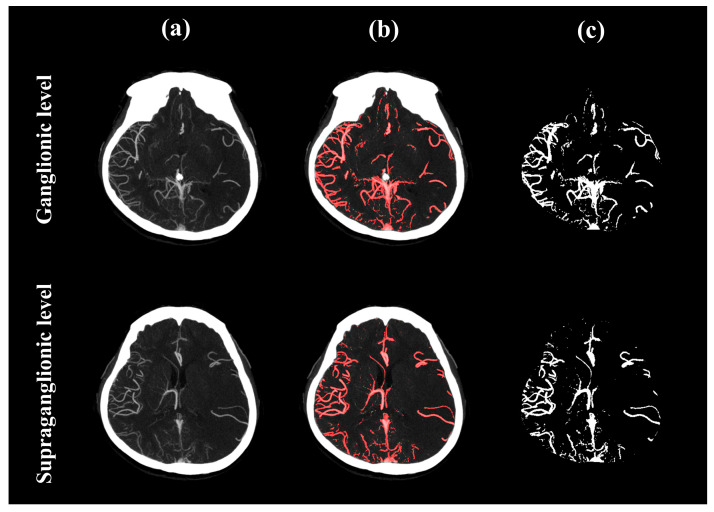
Example images of vessel segmentation. It includes (**a**) original images, (**b**) an overlay of the original images and the segmented vessel using the proposed method, and (**c**) binary images of segmented vessel. The images were obtained from a 69-year-old female patient with left hemisphere occlusion. CTA Menon score = 2.

Vessel segmentation was performed using an adaptive threshold binarization method based on the triangle method [33]. This method selects a binarization threshold based on the image histogram; the schematic diagram in Figure 1 illustrates this method. First, we normalized the histogram peak value and distance from the grayscale value corresponding to the peak to the highest grayscale value (Figure 1a). The normalized histogram (NH) was defined as follows:

(1) $$\;{NH}_{i}=\frac{H_{i}\times \left(255-{\text{Gray\_level}}_{peak}\right)}{H_{peak}}$$

where ${NH}_{i}$ is the normalized value of the number of pixels corresponding to the grayscale value *i* in the image; $H_{i}$ is the number of pixels with the grayscale value *i* in the image; ${\text{Gray\_level}}_{peak}$ is the grayscale value that corresponds to the highest peak value, that is, the grayscale value with the highest frequency; and $H_{peak}$ represents the number of pixels with the highest frequency grayscale. If there are multiple highest values, the rounded mean of the grayscale value is considered to be the highest-frequency grayscale value. Next, a straight line was created by connecting the peak position of the normalized histogram to the position of the highest grayscale, and the vertical Euclidean distance was calculated from the straight line to the corresponding positions of all grayscale values in the range from the peak grayscale to the highest grayscale. The grayscale with the longest distance is selected as the adaptive binarization threshold (Figure 1b). Finally, an AND operation was performed between the brain region image and the binary images obtained by the adaptive threshold to obtain the vessel information required for model training. An example of vessel segmentation is shown in Figure 2.

### 2.3. Collateral Classification Model

**Figure 3:**
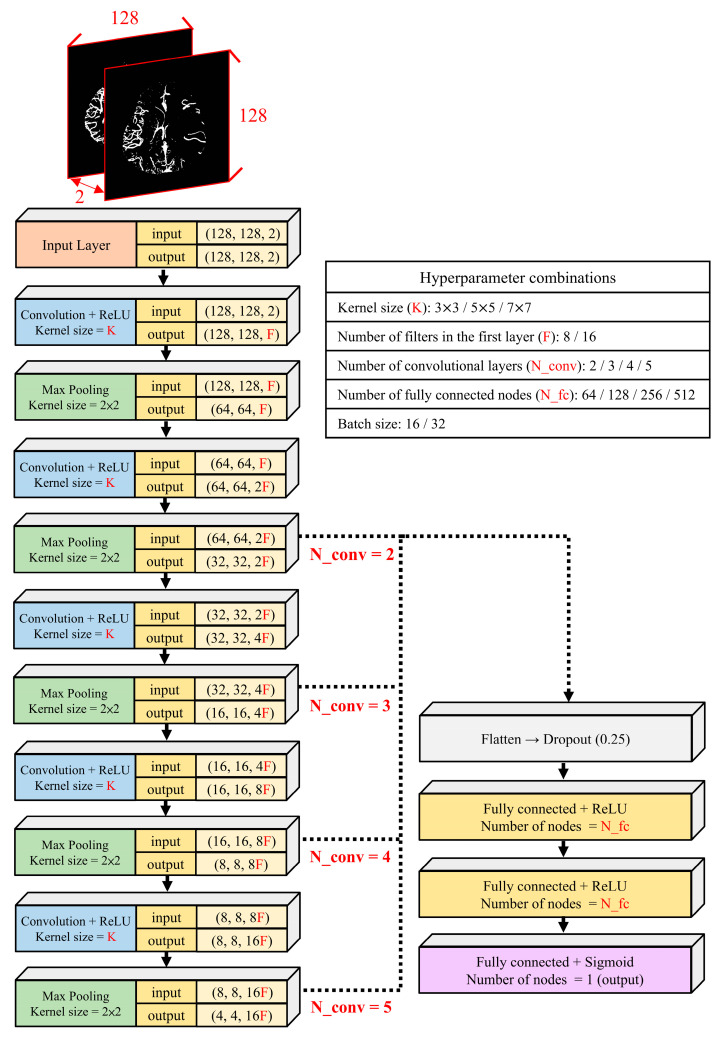
Schematic diagram of CNN model. CNN architectures were constructed with 2 to 5 convolutional layers, using corresponding kernel sizes, number of filters in the first layer, number of fully connected nodes, and batch sizes as part of the hyperparameter combinations.

The implementations were conducted in Python 3.7.16 and TensorFlow 2.4.1, together with relevant open-source libraries. For each patient, two preprocessed binarized vascular morphology images (ganglionic level and supraganglionic level) were selected for training and prediction. Prior to training the collateral classification model, due to insufficient data samples, we performed data augmentation using small-angle rotations and flips on all preprocessed images, increasing the size of the dataset by tenfold. The patient-based split was maintained to avoid data leakage from the same patient. The schematic diagram of CNN model in this study is shown in Figure 3. The ganglionic level and supraganglionic level of each patient were used as model inputs, and the images were resized to 128 × 128. Since the input images only contained binarized vascular morphological information of vascular shape and structure, overly deep models may lead to a higher risk of overfitting. Therefore, the CNN architectures were constructed with 2 to 5 convolutional layers to explore the effects of different numbers of convolutional layers. The number of filters doubled with each additional convolutional layer and other hyperparameter combinations on model performance. Other hyperparameter include kernel size, the number of filters in the first layer, the number of fully connected nodes, and batch size. Specific hyperparameter combinations are shown in Figure 3.

### 2.4. Performance Metrics

In this study, model training for each hyperparameter combination was performed using five-fold cross-validation. All patients were randomly divided into five groups. In each fold, four groups were used for training and validation (with a 3:1 split). The area under the receiver operating characteristic (ROC) curve (AUC) was relatively stable and smooth and was used as the monitor for early stopping. To reflect real clinical scenarios, augmented images were excluded from the test data and only results from the original clinical images were used to evaluate model performance. After repeating the above procedure five times, collateral classification probability of all 157 patients was obtained.

We performed ROC analysis on the classification probability aggregated from the five-fold test data and used the Youden index as the criterion for determining the cut-off point of the ROC curve. The Youden index is defined as follows:

(2) Youden index= Sensitivity+Specificity−1

This study used the optimal Youden index and the corresponding sensitivity, specificity, and accuracy to evaluate model performance. Although the optimal Youden index represents the best classification performance of the model at a specific threshold, a poor Youden index at other cut-off points indicates that the model is highly sensitive to threshold selection, which may lead to unstable results. Therefore, the AUC, which reflects the overall performance of the binary classification task, was also used. The metrics used in this study to evaluate the overall performance of the model are defined as follows:

(3) $$Overall\;performance=\;\frac{optimal\;Youden\;index+AUC}{2}$$

### 2.5. Statistical Analysis

For datasets with 30 or more observations, regardless of the data distribution, it was assumed that the Central Limit Theorem proves the validity of the t-test. For datasets with fewer than 30 observations, the Shapiro–Wilk test was used to assess normality. Since all paired samples contained more than 30 observations, paired *t*-tests were used for their analysis. Independent samples with normally distributed were analyzed using independent t-tests, while independent samples with non-normal distributions were analyzed using the Mann–Whitney U test.

## 3. Results

**Figure 4:**
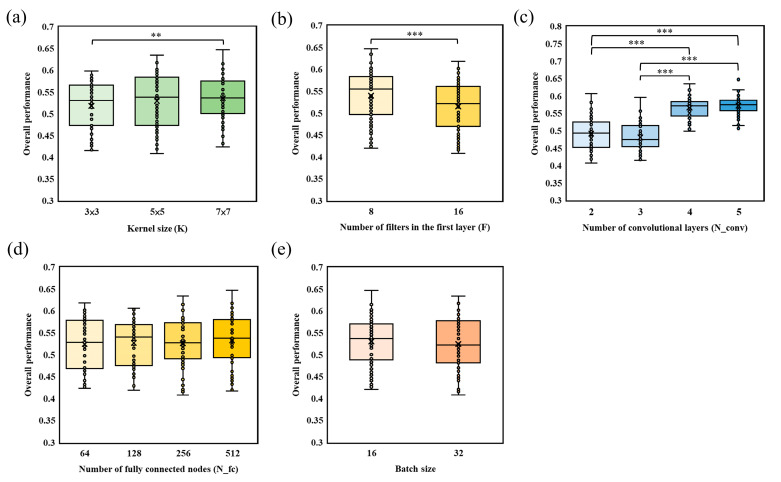
Relationship between CNN hyperparameters and model performance. (**a**) Effect of kernel size on model performance; (**b**) effect of the number of filters in the first layer on model performance; (**c**) effect of the number of convolutional layers on model performance; (**d**) effect of the number of fully connected nodes on model performance; (**e**) effect of batch size on model performance. *p* values were obtained using a paired *t*-test. ** and *** indicate *p* < 0.01 and *p* < 0.001, respectively.

A total of 157 patients were included in this study, of whom 91 were male (61.8%). The median age was 65 years (interquartile range [IQR], 57–74 years). The median baseline NIHSS score, 90-day mRS score, and Menon CTA score were 19 (IQR, 16–22), 4 (IQR, 2–5), and 3 (IQR, 2–4), respectively. A total of 192 models with different hyperparameter combinations were established, and their overall performance was compared across these configurations. The relationships between each CNN hyperparameter and model performance are summarized in Figure 4. Specifically, Figure 4a–e show the effects of kernel size, the number of filters in the first layer, the number of convolutional layers, the number of fully connected nodes, and batch size, respectively.

For kernel size (K) (Figure 4a), as K increased, the dispersion of overall performance became larger and the upper bound of performance also tended to improve. However, a statistically significant difference was observed only between models with K = 3 × 3 and those with K = 7 × 7, indicating that very small and relatively larger kernels may lead to different performance ranges, whereas intermediate settings did not differ significantly. For the number of filters in the first layer (F) (Figure 4b), models initialized with F = 8 filters showed significantly better overall performance than those with F = 16 filters. This suggests that increasing the number of initial filters does not necessarily improve discrimination in this setting and may even be detrimental. For the number of convolutional layers (N_conv) (Figure 4c), models with N_conv = 4 and N_conv = 5 achieved significantly higher overall performance than models with N_conv = 2 or N_conv = 3. In contrast, no significant difference was found between N_conv = 4 and N_conv = 5 or between N_conv = 2 and N_conv = 3, indicating that a minimum network depth is required, but additional layers beyond this threshold yield limited incremental benefit.

Finally, for the number of fully connected nodes (Figure 4d) and batch size (Figure 4e), no statistically significant differences in overall performance were observed among the compared settings. These results suggest that, within the tested ranges, these two hyperparameters have relatively minor influence on model performance compared with kernel size, the number of filters in the first layer, and the number of convolutional layers.

**Figure 5:**
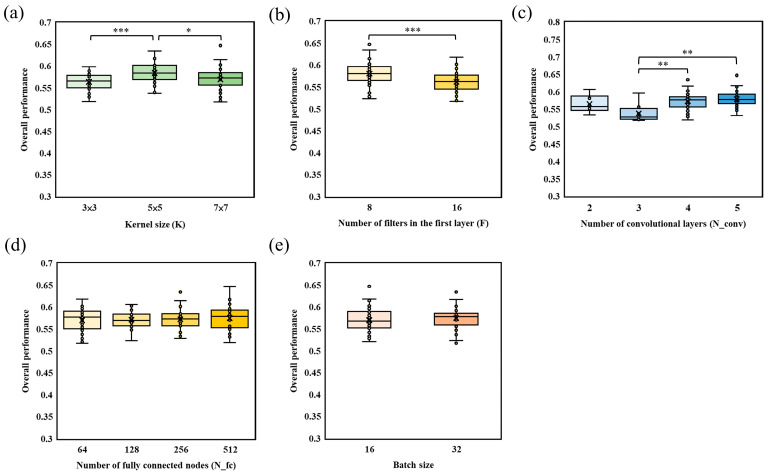
Relationship between CNN hyperparameters and model performance in the subset of models with AUC ≥ 0.7. (**a**) Effect of kernel size on model performance; (**b**) effect of the number of filters in the first layer on model performance; (**c**) effect of the number of convolutional layers on model performance; (**d**) effect of the number of fully connected nodes on model performance; (**e**) effect of batch size on model performance. *p* values were obtained using an independent *t*-test or a Mann–Whitney U test. *, **, and *** indicate *p* < 0.05, *p* < 0.01, and *p* < 0.001, respectively.

To reduce the influence of poorly performing configurations, further analysis was conducted on the subset of models with acceptable discriminative capability (AUC ≥ 0.7). The hyperparameter–performance relationships within this subset are illustrated in Figure 5. When kernel size (K) was examined (Figure 5a), the K = 5 × 5 configuration demonstrated significantly higher overall performance compared with other kernel sizes. However, models with K = 7 × 7 occasionally achieved the highest individual performance values, suggesting that while K = 5 × 5 is generally more stable, larger kernels may still reach superior—but less consistent—results under specific combinations. Regarding the number of filters in the first layer (F) (Figure 5b), models initialized with F = 8 consistently outperformed those initialized with F = 16, reinforcing trends observed in the full model set and implying that excessive early feature expansion may hinder performance rather than enhance it.

A clear distinction emerged when comparing convolutional depth (N_conv) (Figure 5c). Only a small number of models with N_conv = 2 or N_conv = 3 remained in the AUC ≥ 0.7 subset (*n* = 6 and *n* = 8, respectively), whereas most configurations with N_conv = 4 or N_conv = 5 reached this performance threshold (*n* = 43 and *n* = 41, respectively). This finding suggests that shallower CNN architectures generally lack sufficient capacity for effective collateral feature representation, while deeper models reliably produce acceptable discriminative power. Nevertheless, as with kernel size, the performance difference between N_conv = 4 and N_conv = 5 was not statistically significant, indicating that four convolutional layers may serve as a practical structural baseline without requiring further depth. In contrast, neither the number of fully connected nodes nor batch size (Figure 5d,e) produced noticeable performance shifts within the AUC-filtered subset, supporting the conclusion that these two hyperparameters exert comparatively minimal influence relative to kernel size, initial channel count, and convolutional depth.

**Figure 6:**
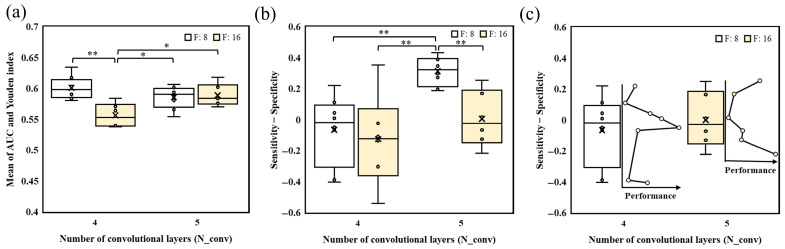
Performance of model subset using 5 × 5 kernel size (AUC ≥ 0.7). (**a**) Overall performance; (**b**) difference between sensitivity and specificity (sensitivity—specificity); (**c**) relationship between sensitivity—specificity and overall performance. *p* values were obtained using an independent *t*-test. * and ** indicate *p* < 0.05 and *p* < 0.01.

Building on these findings, we further examined performance behavior under the most promising hyperparameter region (AUC ≥ 0.7). Because kernel size, filter count, and convolutional depth were identified as key determinants, detailed comparisons were conducted using the best-performing kernel configuration (K = 5 × 5), stratified by N_conv and F (Figure 6). When N_conv = 4 and F = 8, the models showed the highest median performance and a stable distribution range (Figure 6a). Performance tended to balance sensitivity and specificity (Figure 6c), indicating that four convolutional layers with moderate filter capacity allow the network to extract generalizable vascular patterns without overwhelming parametric complexity. In contrast, increasing depth to N_conv = 5 with the same initial filter count (F = 8) shifted the model toward higher sensitivity (Figure 6b), suggesting that deeper feature abstraction improved recognition of collateral-positive patterns but may also amplify false-positive responses when morphological features become diffused. A different trend emerged when filter count increased. With N_conv = 4 and F = 16, overall performance declined significantly. This suggests that higher channel capacity introduced redundant parameters, which the four-layer network was unable to effectively regulate. However, when both depth and filter number were increased together (N_conv = 5, F = 16), performance recovered noticeably, and these models exhibited a greater inclination toward specificity—likely because deeper layers compensated for parameter inflation by enabling more selective high-level feature abstraction. Within this configuration, the highest-performing models were those biased toward specificity, indicating improved discrimination of poor collateral patterns (Figure 6c). Taken together, these results demonstrate that different hyperparameter combinations do not merely affect absolute accuracy but also actively shape performance orientation. The (K = 5 × 5, F = 8, N_conv = 4) configuration favored balanced decision behavior, (K = 5 × 5, F = 8, N_conv = 5) biased toward sensitivity, and (K = 5 × 5, F = 16, N_conv = 5) promoted specificity. This reveals a tunable model architecture framework, allowing the classifier to be adjusted toward sensitivity or specificity depending on EVT decision requirements.

**Figure 7:**
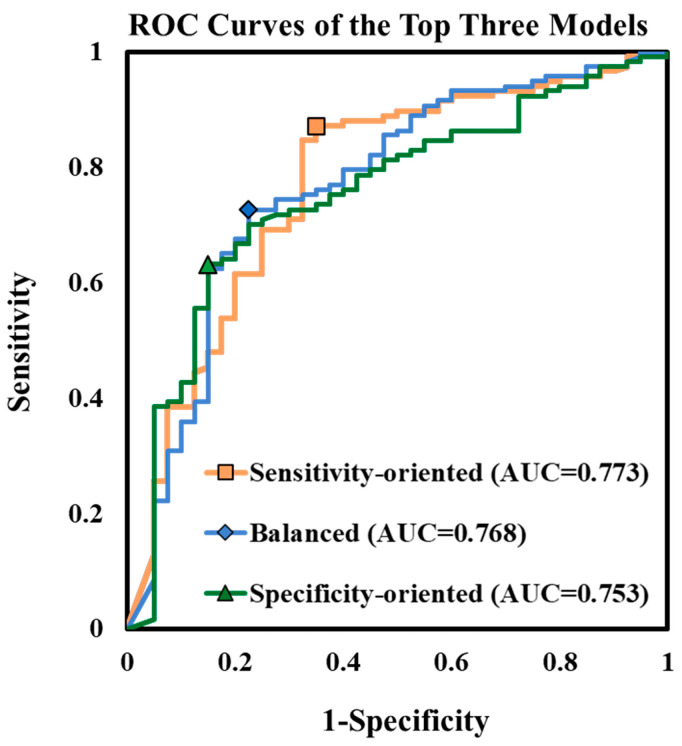
ROC curves of the top three performing models, including a sensitivity-oriented model (AUC = 0.773), a balanced model (AUC = 0.768), and a specificity-oriented model (AUC = 0.753). The optimal Youden index for each model is indicated by square, diamond, and triangle markers, respectively.

Finally, the three best-performing models across all tested hyperparameter combinations were identified and compared (Figure 7). In addition, we also compared the model performance under imbalance conditions in a recent non-commercial software study (Table 1). In H. Kuang et al.’s study [28], the numbers of positive (non-poor collateral) and negative (poor collateral) samples were 121 and 33, respectively, which is slightly more imbalanced than in our study (117 good collateral vs. 40 poor collateral). Their results showed the highest sensitivity but the lowest specificity. Although this resulted in higher accuracy, the overall performance, AUC, and optimal Youden index were similar to those of our sensitivity-oriented model, and good performance was achieved only for the majority class. In contrast, the three best-performing models in our study represented distinct behavioral tendencies, allowing classification preference to shift between sensitivity, balance, and specificity depending on clinical requirements. The sensitivity-oriented model (K = 7 × 7, F = 8, N_conv = 5) achieved the highest sensitivity (87.18%) with an AUC of 0.773 but exhibited lower specificity (65.00%), making it suitable for scenarios prioritizing detection of salvageable patients. The balanced model (K = 5 × 5, F = 8, N_conv = 4) demonstrated comparable AUC (0.768) with more even performance across sensitivity (72.65%) and specificity (77.50%), suggesting broad applicability as a general decision model. In contrast, the third model (K = 5 × 5, F = 16, N_conv = 5) showed the highest specificity (85.00%) with moderate sensitivity (63.25%), reflecting a specificity-driven decision bias ideal for conservative treatment selection or for reducing unnecessary EVT risk. Notably, the hyperparameter patterns observed in these three models were consistent with the feature–behavior relationships derived from Figure 6. Deeper architectures with moderate filter counts favored sensitivity, moderate depth with fewer filters yielded balanced classification, and deeper networks with larger filter capacity produced specificity-oriented responses. This convergence across analyses confirms that model behavior in CTA collateral classification can be systematically controlled through hyperparameter configuration rather than obtained only by chance or trial-and-error tuning. These findings demonstrate the feasibility of designing clinically adjustable AI models—capable of operating in sensitivity-oriented, specificity-oriented, or balanced modes—based solely on architectural selection. This adaptability has direct translational relevance, enabling deployment tailored to triage intention, resource constraints, or risk tolerance in EVT decision workflows.

## 4. Discussion

This study systematically evaluated 192 CNN architectures for CTA-based collateral classification in AIS and demonstrated that hyperparameter interactions influence not only predictive accuracy but also model behavioral orientation. Kernel size, initial filter count, and convolutional depth emerged as the primary determinants governing performance orientation. Rather than being a by-product of random hyperparameter search, these results indicate that sensitivity-oriented, specificity-oriented, and balanced behaviors are architecturally programmable outcomes. Findings from the AUC-qualified subset indicate that model behavior is not solely determined by accuracy but by how capacity is distributed across architectural depth and channel width. Networks with four convolutional layers and eight initial filters reached a stable representational equilibrium, minimizing bias toward sensitivity or specificity. Increasing depth to five layers without expanding channel width redirected the model toward sensitivity, implying that deeper spatial abstraction improves recognition of well-perfused collateral patterns, though at the cost of susceptibility to false-positive amplification. Conversely, expanding capacity through both depth and filters enabled a more discriminative response, particularly favoring specificity when collateral vessels were sparse. These trends aligned with the three observed performance archetypes—sensitivity-oriented (K = 7 × 7, F = 8, N_conv = 5), balanced (K = 5 × 5, F = 8, N_conv = 4), and specificity-oriented (K = 5 × 5, F = 16, N_conv = 5)—reinforcing that network behavior can be intentionally shaped rather than passively observed.

The clinical relevance of controllable model bias is substantial. In EVT decision support, the consequences of misclassification differ fundamentally between sensitivity and specificity perspectives. Sensitivity-oriented models may be advantageous during early triage when maximizing salvageability is critical, especially for patients who may otherwise be excluded from reperfusion. Conversely, specificity-oriented configurations may help prevent unnecessary thrombectomy in borderline cases or in environments with limited procedural capacity. The balanced configuration represents a pragmatic default, offering stable operation across heterogeneous populations without skewing decision intent. This framework reflects a conceptual shift—from static prediction toward architecture-guided, decision-adaptive collateral AI—enabling systems to be selected or tuned according to therapeutic strategy rather than fixed AUC metrics alone.

Another contribution lies in the streamlined single-phase MIP-based pipeline. Most existing automated collateral systems rely on 3D CTA or multiphase imaging, requiring computational resources or proprietary licensing [20,21,26,27], which may hinder adoption outside major centers. In contrast, the vessel-extracted 2D CNN developed here uses basic image processing algorithms and standard CNN architectures and is implemented entirely using open-source frameworks, which can be deployed on common computing platforms without specialized hardware. This allows it to be easily integrated into existing PACS and hospital information systems without requiring proprietary interfaces, commercial software licenses, or additional service fees. These characteristics enhance the clinical applicability of the method by lowering deployment cost, increasing system compatibility, and enabling transparent and reproducible model behavior. In a clinical workflow, the proposed method is intended to serve as a decision support or pre-screening tool that assists clinicians by providing collateral classification results of CTA images of patients with AIS due to large-vessel occlusion of the anterior circulation. Nevertheless, over-reliance on sensitivity-oriented models may increase false-positive classifications in borderline cases, which could in turn impose additional burdens on downstream clinical workflows and resource utilization. In contrast, specificity-oriented models may underestimate potentially salvageable tissue if applied without expert review. Therefore, these models are not intended to be used as standalone decision-making tools for reperfusion therapy selection, and their outputs should be interpreted in conjunction with clinical judgment and established diagnostic criteria rather than replacing expert interpretation.

Medical imaging datasets, including stroke CTA, often suffer from limited sample size and imbalanced distribution, making balanced sampling difficult and predisposing models to majority-class bias. Prior work has shown that models trained under imbalance may generalize poorly to minority categories [28,34], which is particularly relevant for poor-collateral patients. As collateral status directly reflects salvageability [35], over-emphasis on the majority class could misinform treatment decisions. The present findings provide a data-driven basis for selecting architectures that maintain stable behavior across imbalance conditions or can be tuned according to clinical need. This work establishes a mechanistic understanding of how architectural elements modulate collateral classification behavior and presents a practical path toward tailored decision support—whether sensitivity-first, specificity-first, or performance-neutral. With external validation and prospective clinical testing, architecture-adaptive AI may enhance EVT triage precision, support individualized stroke management, and expand access to automated collateral assessment across different levels of medical infrastructure.

This study has several limitations. First, the relatively small single-center cohort and its retrospective design may lead to data bias and insufficient heterogeneity, thereby limiting the generalizability of the findings. Second, collateral status was assessed by two neuroradiologists according to Menon’s criteria, which may introduce subjective bias. Third, the timing of CTA acquisition was not considered in this study, and previous studies have indicated that the lack of temporal information in single-phase CTA may result in biased assessment of collateral status [30]. Therefore, the proposed approach should be interpreted as an exploratory tool rather than a definitive solution for clinical decision-making at this stage. Finally, due to computational constraints, the experiments were conducted using only a single random seed. Future work will perform multiple calculations with different random seeds to further verify the robustness of the current findings under various data-splitting conditions.

## 5. Conclusions

This study developed a CNN model for automatic collateral classification from CTA images of patients with AIS and explored the impact of different hyperparameter combinations on model performance trends. We demonstrated that model performance is not only determined by overall architecture strength but can be intentionally shaped toward sensitivity, specificity, or balance through the manipulation of kernel size, filter capacity, and convolutional depth. The three resulting optimal models reflected this tunability—representing sensitivity-first, specificity-first, and neutral decision behaviors—and collectively revealed a structural mechanism by which model orientation can be engineered rather than empirically discovered. By leveraging single-phase MIP vessel morphology, the proposed framework reduces computational requirements while maintaining clinically meaningful performance, providing a feasible pathway for deployment in diverse healthcare environments. These findings support the development of decision-adaptive collateral AI, allowing clinicians to select or integrate models based on therapeutic goals such as maximizing salvage detection or reducing unnecessary interventions.

Future work will focus on multi-center validation, robustness evaluation under multi-seed training, and strategies to mitigate class imbalance, including focal losses and ensemble fusion of sensitivity- and specificity-driven networks. With further validation and workflow integration, the proposed adaptive architecture has the potential to enhance EVT triage precision and support more individualized management strategies for acute ischemic stroke.

## Figures and Tables

**Table 1:** Performance comparison of the top three models.

Model (K, F, N_conv)	Overall Performance	AUC	Optimal Youden Index	Sensitivity	Specificity	Accuracy
Sensitivity-oriented model (K = 7 × 7, F = 8, N_conv = 5)	0.647	0.773	0.522	87.18%	65.00%	81.53%
Balanced model (K = 5 × 5, F = 8, N_conv = 4)	0.635	0.768	0.501	72.65%	77.50%	73.89%
Specificity-oriented model (K = 5 × 5, F = 16, N_conv=5)	0.618	0.753	0.482	63.25%	85.00%	68.79%
H. Kuang et al. [12]	0.649	0.766	0.532	92.56%	60.61%	85.71%

## Data Availability

Data supporting the findings of this study are available from the corresponding author upon reasonable request and with institutional approval. The data are not publicly available due to the approved protocol not covering public data sharing.

## Abbreviations

The following abbreviations are used in this manuscript:

EVT	Endovascular thrombectomy
AIS	Acute ischemic stroke
CTA	Computed tomography angiography
CNN	Convolutional neural networks
NIHSS	National Institutes of Health Stroke Scale
mRS	modified Rankin Scale
PACS	Picture Archiving and Communication System
IQR	Interquartile range
ROC	Receiver operating characteristic
AUC	Area under the ROC curve
K	Kernel sizes
F	Number of filters in the first layer
N_conv	Number of convolutional layers
N_fc	Number of fully connected nodes

## References

[1] Goyal M., Menon B.K., Van Zwam W.H., Dippel D.W., Mitchell P.J., Demchuk A.M., Dávalos A., Majoie C.B., van Der Lugt A., De Miquel M.A. (2016). Endovascular thrombectomy after large-vessel ischaemic stroke: A meta-analysis of individual patient data from five randomised trials. Lancet.

[2] Berkhemer O.A., Fransen P.S., Beumer D., Van Den Berg L.A., Lingsma H.F., Yoo A.J., Schonewille W.J., Vos J.A., Nederkoorn P.J., Wermer M.J. (2015). A randomized trial of intraarterial treatment for acute ischemic stroke. N. Engl. J. Med..

[3] Goyal M., Demchuk A.M., Menon B.K., Eesa M., Rempel J.L., Thornton J., Roy D., Jovin T.G., Willinsky R.A., Sapkota B.L. (2015). Randomized assessment of rapid endovascular treatment of ischemic stroke. N. Engl. J. Med..

[4] Campbell B.C., Mitchell P.J., Kleinig T.J., Dewey H.M., Churilov L., Yassi N., Yan B., Dowling R.J., Parsons M.W., Oxley T.J. (2015). Endovascular therapy for ischemic stroke with perfusion-imaging selection. N. Engl. J. Med..

[5] Saver J.L., Goyal M., Bonafe A., Diener H.-C., Levy E.I., Pereira V.M., Albers G.W., Cognard C., Cohen D.J., Hacke W. (2015). Stent-retriever thrombectomy after intravenous t-PA vs. t-PA alone in stroke. N. Engl. J. Med..

[6] Jovin T.G., Chamorro A., Cobo E., de Miquel M.A., Molina C.A., Rovira A., San Román L., Serena J., Abilleira S., Ribó M. (2015). Thrombectomy within 8 hours after symptom onset in ischemic stroke. N. Engl. J. Med..

[7] Bang O.Y., Goyal M., Liebeskind D.S. (2015). Collateral circulation in ischemic stroke: Assessment tools and therapeutic strategies. Stroke.

[8] Berkhemer O.A., Jansen I.G., Beumer D., Fransen P.S., Van Den Berg L.A., Yoo A.J., Lingsma H.F., Sprengers M.E., Jenniskens S.F., Lycklama à Nijeholt G.J. (2016). Collateral status on baseline computed tomographic angiography and intra-arterial treatment effect in patients with proximal anterior circulation stroke. Stroke.

[9] Sinha A., Stanwell P., Beran R.G., Calic Z., Killingsworth M.C., Bhaskar S.M. (2021). Stroke aetiology and collateral Status in acute ischemic stroke patients receiving reperfusion therapy—A meta-analysis. Neurol. Int..

[10] de Havenon A., Haynor D.R., Tirschwell D.L., Majersik J.J., Smith G., Cohen W., Andre J.B. (2017). Association of collateral blood vessels detected by arterial spin labeling magnetic resonance imaging with neurological outcome after ischemic stroke. JAMA Neurol..

[11] Sheth K.N., Terry J.B., Nogueira R.G., Horev A., Nguyen T.N., Fong A.K., Gandhi D., Prabhakaran S., Wisco D., Glenn B.A. (2013). Advanced modality imaging evaluation in acute ischemic stroke may lead to delayed endovascular reperfusion therapy without improvement in clinical outcomes. J. Neurointerv. Surg..

[12] McVerry F., Liebeskind D., Muir K. (2012). Systematic review of methods for assessing leptomeningeal collateral flow. Am. J. Neuroradiol..

[13] Maas M.B., Lev M.H., Ay H., Singhal A.B., Greer D.M., Smith W.S., Harris G.J., Halpern E., Kemmling A., Koroshetz W.J. (2009). Collateral vessels on CT angiography predict outcome in acute ischemic stroke. Stroke.

[14] Miteff F., Levi C.R., Bateman G.A., Spratt N., McElduff P., Parsons M.W. (2009). The independent predictive utility of computed tomography angiographic collateral status in acute ischaemic stroke. Brain.

[15] Tan I., Demchuk A., Hopyan J., Zhang L., Gladstone D., Wong K., Martin M., Symons S., Fox A., Aviv R. (2009). CT angiography clot burden score and collateral score: Correlation with clinical and radiologic outcomes in acute middle cerebral artery infarct. Am. J. Neuroradiol..

[16] Lu Q., Zhang H., Cao X., Fu J., Pan Y., Zheng X., Wang J., Geng D., Zhang J. (2022). Quantitative collateral score for the prediction of clinical outcomes in stroke patients: Better than visual grading. Front. Neurosci..

[17] Boers A., Barros R.S., Jansen I., Berkhemer O., Beenen L., Menon B.K., Dippel D., van der Lugt A., van Zwam W., Roos Y. (2018). Value of quantitative collateral scoring on CT angiography in patients with acute ischemic stroke. Am. J. Neuroradiol..

[18] Boers A., Berkhemer O., Slump C., Van Zwam W., Roos Y., van der Lugt A., van Oostenbrugge R., Yoo A.J., Dippel D., Marquering H. (2017). Topographic distribution of cerebral infarct probability in patients with acute ischemic stroke: Mapping of intra-arterial treatment effect. J. Neurointerv. Surg..

[19] Uniken Venema S.M., Wolff L., van den Berg S.A., Reinink H., Luijten S.P., Lingsma H.F., Marquering H.A., Boers A.M., Bot J., Hammer S. (2022). Time since stroke onset, quantitative collateral score, and functional outcome after endovascular treatment for acute ischemic stroke. Neurology.

[20] Wolff L., Uniken Venema S.M., Luijten S.P., Hofmeijer J., Martens J.M., Bernsen M.L.E., van Es A.C., van Doormaal P.J., Dippel D.W., van Zwam W. (2022). Diagnostic performance of an algorithm for automated collateral scoring on computed tomography angiography. Eur. Radiol..

[21] Grunwald I.Q., Kulikovski J., Reith W., Gerry S., Namias R., Politi M., Papanagiotou P., Essig M., Mathur S., Joly O. (2019). Collateral automation for triage in stroke: Evaluating automated scoring of collaterals in acute stroke on computed tomography scans. Cerebrovasc. Dis..

[22] Dou Q., Chen H., Yu L., Qin J., Heng P.-A. (2016). Multilevel contextual 3-D CNNs for false positive reduction in pulmonary nodule detection. IEEE Trans. Biomed. Eng..

[23] Monkam P., Qi S., Xu M., Han F., Zhao X., Qian W. (2018). CNN models discriminating between pulmonary micro-nodules and non-nodules from CT images. Biomed. Eng. Online.

[24] Hafeez H.A., Elmagzoub M.A., Abdullah N.A.B., Al Reshan M.S., Gilanie G., Alyami S., Hassan M.U., Shaikh A. (2023). A CNN-model to classify low-grade and high-grade glioma from mri images. IEEE Access.

[25] Ali N.H., Abdullah A.R., Saad N.M., Muda A.S. (2023). Collateral circulation classification based on cone beam computed tomography images using ResNet18 convolutional neural network. Int. J. Adv. Comput. Sci. Appl..

[26] Su J., Wolff L., van Es A.C.M., Van Zwam W., Majoie C., Dippel D.W., Van Der Lugt A., Niessen W.J., Van Walsum T. (2020). Automatic collateral scoring from 3D CTA images. IEEE Trans. Med. Imaging.

[27] Fortunati V., Su J., Wolff L., van Doormaal P.-J., Hofmeijer J., Martens J., Bokkers R.P., van Zwam W.H., van der Lugt A., van Walsum T. (2024). Siamese model for collateral score prediction from computed tomography angiography images in acute ischemic stroke. Front. Neuroimaging.

[28] Kuang H., Wan W., Wang Y., Wang J., Qiu W. (2023). Automated collateral scoring on CT angiography of patients with acute ischemic stroke using hybrid CNN and transformer network. Biomedicines.

[29] Sahoo A., Abdalkader M., Yamagami H., Huo X., Sun D., Jia B., Weyland C.S., Diana F., Kaliaev A., Klein P. (2023). Endovascular therapy for acute stroke: New evidence and indications. J. Neuroendovasc. Ther..

[30] Menon B.K., d’Esterre C.D., Qazi E.M., Almekhlafi M., Hahn L., Demchuk A.M., Goyal M. (2015). Multiphase CT angiography: A new tool for the imaging triage of patients with acute ischemic stroke. Radiology.

[31] Chang C.-H., Ku C.-M., Ger T.-R., Lin W.-P. (2025). Fractal-Based Quantitative Collateral Assessment for Thrombectomy Candidate Selection in Acute Ischemic Stroke: A Preliminary Study. Diagnostics.

[32] Won Y.-D., Kim J.-M., Cheong J.-H., Ryu J.-I., Koh S.-H., Han M.-H. (2021). Effect of possible osteoporosis on parenchymal-type hemorrhagic transformation in patients with cardioembolic stroke. J. Clin. Med..

[33] Zack G.W., Rogers W.E., Latt S.A. (1977). Automatic measurement of sister chromatid exchange frequency. J. Histochem. Cytochem..

[34] Huang C.-C., Chiang H.-F., Hsieh C.-C., Chou C.-L., Jhou Z.-Y., Hou T.-Y., Shaw J.-S. (2023). Using deep-learning-based artificial intelligence technique to automatically evaluate the collateral status of multiphase cta in acute ischemic stroke. Tomography.

[35] Bang O.Y., Saver J.L., Buck B.H., Alger J.R., Starkman S., Ovbiagele B., Kim D., Jahan R., Duckwiler G.R., Yoon S.R. (2008). Impact of collateral flow on tissue fate in acute ischaemic stroke. J. Neurol. Neurosurg. Psychiatry.

